# Research on the Resistance Performance and Damage Deterioration Model of Fiber-Reinforced Gobi Aggregate Concrete

**DOI:** 10.3390/ma17102291

**Published:** 2024-05-12

**Authors:** Tuo Lei, Hai Bai, Lei Li

**Affiliations:** School of Civil Engineering, Chang’an University, Xi’an 710061, China; leituo616@163.com (T.L.); gxyfzgxyfz@163.com (L.L.)

**Keywords:** freeze–thaw cycle, fiber-reinforced Gobi concrete, mass loss rate, relative dynamic elastic modulus, freeze–thaw damage model

## Abstract

Concrete prepared using Gobi sand and gravel instead of ordinary sand and gravel is referred to as Gobi concrete. In order to explore the effect of fibers on the frost resistance of Gobi concrete, as well as to enhance the service life of Gobi aggregate concrete in Northwest China, experiments were conducted with fiber types (polypropylene fibers, basalt fibers, polypropylene–basalt fibers) and fiber volume fractions (0%, 0.1%, 0.2%, 0.3%) as variable parameters. This study investigated the surface morphology, mass loss rate, and relative dynamic elastic modulus of fiber-reinforced Gobi concrete after different freeze–thaw cycles (0, 25, 50, 75, 100). Corresponding frost damage deterioration models were proposed. The results indicate that fibers have a favorable effect on the anti-peeling performance, mass loss rate, and dynamic elastic modulus of Gobi aggregate concrete. The improvement levels of different fiber types are in the following order: 0.1% basalt-polypropylene fibers, 0.2% polypropylene fibers, and 0.3% basalt fibers. Compared to Gobi concrete exposed to natural environmental conditions, the freeze–thaw cycle numbers increased by 343, 79, and 69 times, respectively. A quadratic polynomial damage model for fiber-reinforced Gobi concrete, using relative dynamic elastic modulus as the damage variable, was established and demonstrated good predictive performance.

## 1. Introduction

Northwest China, as a key node in the “Belt and Road” initiative, is experiencing explosive growth in its infrastructure construction. However, there is a sharp increase in the demand for construction materials. Among the distinctive resources in the Northwestern region, Gobi aggregate has become a research focus due to its excellent characteristics such as good surface roundness, appropriate porosity, low moisture content, and high shear strength [1]. Currently, research on Gobi aggregate primarily focuses on the field of mine filling. For instance, Yang et al. [2] examined the impact of the water–cement ratio and Gobi aggregate substitution rate on the fluid pressure drop of whole tailings and Gobi aggregate paste, utilizing a validated friction coefficient model. Deng et al. [3], through experimental studies, clarified that a reasonable ratio of tailings to Gobi aggregate in backfill material is 1:4. Given the current context of large-scale construction in Northwest China, if Gobi aggregate is used to replace traditional aggregate in the preparation of concrete, it can address the issues of the shortage of concrete raw materials, high construction costs, and extended construction periods. Zhang et al. [4] conducted tests on the Gobi concrete ratio and found that Gobi concrete meets the design requirements in terms of strength, seepage resistance, and frost resistance, thus demonstrating the feasibility of using Gobi material in concrete preparation. Furthermore, Farouk et al. [5] prepared Gobi concrete using high-performance cement instead of ordinary silicate cement, revealing that high-performance cement can overcome the poor concrete performance-related issues caused by the high mud content of Gobi gravel.

In cold regions, concrete structures are often exposed to low-temperature environments, and freeze–thaw cycles can exacerbate the expansion of initial cracks within concrete and induce the formation of new cracks [6,7]. However, this problem can be alleviated through the incorporation of basalt fibers. The principle behind this is that the bridging effect of fibers slows down the development of microcracks within concrete, impeding the occurrence and progression of macrocracks, and it is considered one of the most effective approaches to enhance concrete frost resistance [8,9].

Tensile strength holds a crucial position in resisting frost heave and sulfate attack. Consequently, fiber-modified concrete exhibits excellent performance under freeze–thaw cycles [10]. Although steel fibers are capable of enhancing the tensile strength and toughness of concrete, they often present issues such as susceptibility to agglomeration, inadequate compatibility, challenges in pumping, construction complexities, and elevated costs. In contrast, polypropylene fibers and basalt fibers exhibit superior performance in addressing these concerns. Polypropylene fibers can effectively improve the durability, crack resistance, ductility, compressive strength, and splitting tensile strength of concrete materials [8,11]. Basalt fibers can effectively enhance the ductility, tensile strength, crack resistance, and durability of concrete [12,13]. Recently, scholars both domestically and internationally have conducted research on the frost resistance of polypropylene fiber concrete and basalt fiber concrete, with the following findings: Yan et al. [14] conducted experimental research on the frost resistance of polypropylene fiber concrete from the perspectives of material properties, material mix ratios, and the number of freeze–thaw cycles. The study showed that the addition of air-entraining agents can improve the frost resistance of polypropylene concrete. Richardson et al. [15] and Ren et al. [16] pointed out that polypropylene fibers enhance the frost resistance of concrete, primarily attributed to their bridging effect, which effectively mitigates damage caused by freeze–thaw cycles. Jie et al. [17] studied the influence of different basalt fiber contents on the frost resistance of recycled concrete, indicating that basalt fibers can slow down the freeze–thaw damage of recycled concrete, with the best improvement effect observed at a content of 1.2 kg/m^3^. Xue et al. [18] investigated the fracture performance of basalt fiber concrete with different fiber contents and aspect ratios. The study revealed that basalt fibers significantly enhance the fracture performance of concrete, including fracture toughness, fracture energy, and ductility index. Zhang et al. [19] formulated two freeze–thaw cycle damage models, utilizing thermodynamic and attenuation theories as the foundation, to describe the intricate freeze–thaw cycle process undergone by basalt fiber-reinforced concrete.

Although a lot of studies have been conducted on the frost resistance of fiber-reinforced ordinary concrete, there is a gap in the research on the frost resistance of fiber-reinforced Gobi concrete. First of all, due to the characteristics of Gobi aggregates with high mud contents, large proportions of clay mineral content, and high water absorption rates [20], it is possible that Gobi concrete generates greater hydraulic pressure and osmotic pressure than ordinary concrete during freezing, which makes it easier for Gobi concrete to reach the critical water saturation threshold for freeze–thaw damage [21]. Secondly, although normal concrete can also be improved by the addition of fibers to enhance its durability, the enhancement achieved through fibers may not be as significant as in the case of Gobi concrete due to the relatively good inherent properties of normal concrete. Furthermore, although the inclusion of fibers may add some cost, the improved performance and enhanced durability that they bring can render Gobi concrete competitive in a broader range of applications. Finally, the establishment of a freeze–thaw damage model for fiber-reinforced Gobi concrete is valuable for predicting the life and evaluating the durability of Gobi concrete structures in Northwest China.

Therefore, the present study aims to investigate the damage and deterioration mechanisms of fiber-reinforced Gobi aggregate concrete under freeze–thaw cycles by incorporating different types (polypropylene fibers, basalt fibers, polypropylene–basalt fibers) and volumetric contents (0%, 0.1%, 0.2%, 0.3%) of fibers. Subsequently, utilizing damage mechanics theory, deterioration models are established based on relative dynamic elastic modulus as a parameter. This study seeks to provide a reference basis for the promotion and application of Gobi aggregate concrete in Northwest China.

## 2. Materials and Methodology

### 2.1. Materials

**Figure 1:**
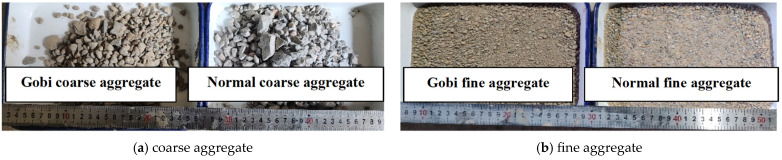
The morphology of aggregate.

The Gobi aggregate used in the experiment was obtained from the excavation of Gobi material at Urumqi International Airport North Area Expansion Project in Xinjiang, China. After screening, coarse and fine aggregates were obtained, and their various physical properties are listed in Table 1. Figure 1 shows the apparent morphology of Gobi aggregate and ordinary aggregate. It can be seen that Gobi aggregate has a clay color and is more rounded in shape than ordinary aggregates. Sea Snail brand P·O42.5R ordinary Portland cement (Anhui Conch Cement Co. Ltd., Wuhu, China) was utilized. Fly ash was sourced from Yuanyuan Jing Shui, classified as Class I fly ash. Mixing water was natural tap water. The physical properties of polypropylene fibers and basalt fibers are detailed in Table 2.

X-ray diffractometer (Rigaku Corporation, Tokyo, Japan) was used to test the mineral composition of Gobi materials at a rate of 8°/min. The test results showed that the main components of Gobi aggregates are plagioclase feldspar, quartz, and clay, of which the relative percentages of mineral components of the studied Gobi coarse aggregates were 45.5%, 32.6%, and 18.8%, respectively, and the relative percentages of mineral components of the studied Gobi fine aggregates were 26.6%, 41.2%, and 20.5%. From the results of the analyses, it is clear that the highest percentage of clay content is found in the Gobi aggregate. Further testing of the mineral composition of clay showed that the mineral fraction of clay is dominated by monazite and illite (listed in Table 3).

### 2.2. Mix Proportions and Specimen Preparations

Ten sets of concrete mix proportions were designed with fiber types (polypropylene fibers, basalt fibers, polypropylene fibers, and basalt fibers) and fiber volume fractions (0%, 0.1%, 0.2%, 0.3%) as variable parameters, as shown in Table 4. All specimens were tagged with a label as follows: PC, BF, PF, and BF + PF represent the Gobi concrete without fiber-, basalt fiber-, polypropylene fiber-, and basalt-polypropylene hybrid fiber-reinforced Gobi concrete, respectively; the numbers of 0.1, 0.2, and 0.3 indicate the volume fractions of fibers equal to 0.1%, 0.2%, and 0.3%, respectively. For instance, BF-0.1 denotes the basalt fiber-reinforced concrete with a fiber volume fraction of 0.1%.

The mixing process of concrete was as follows: firstly, the dried coarse aggregate and fine aggregate were mixed for 30 s; then, cement and aggregates were mixed for 1 min; subsequently, the water was added and mixing continued for 2 min. Finally, the fibers were added and the mixture was continued for 2 min until all the materials were well mixed. After all materials were well mixed, specimens were cast and compacted in molds on the vibration table, and covered with polyethylene sheets on cast surfaces. The experimental procedures and data processing followed the specifications outlined in the “Code for Design of Concrete Mix Proportions” [22].

### 2.3. Procedures and Methods

**Figure 2:**
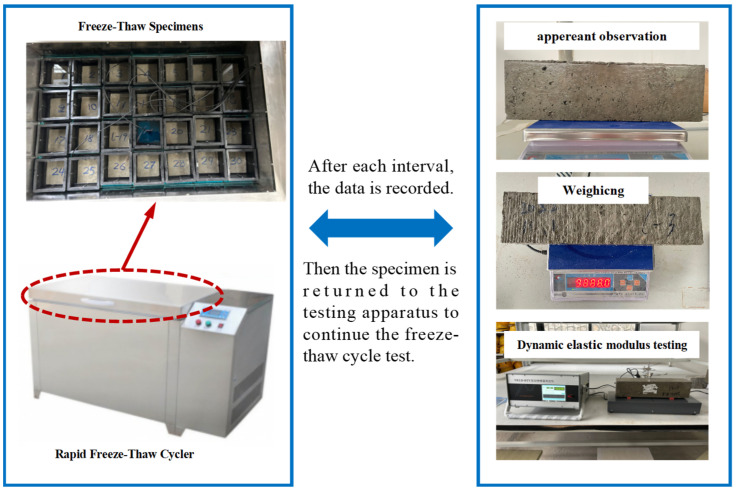
The procedure of the freeze–thaw cycle test.

After a 24-day curing period, the specimens were removed and soaked in water for another 4 days, ensuring a water level exceeding the specimen’s height by at least 20 mm for complete saturation. The specimens used in the experiment measured 100 mm × 100 mm × 400 mm, with each experimental group comprising three specimens. Each freeze–thaw cycle was set to last for 4 h. During the freezing stage, the central temperature of specimen was meticulously controlled within the range of −18 ± 2 °C. Similarly, during the melting stage, the central temperature of the specimen was maintained within the range of 5 ± 2 °C. The mass and dynamic elastic modulus of the specimens were measured after every 25 freeze–thaw cycles, and the freeze–thaw damage morphology on the surface of the specimens was observed. In the experiment, the specimen was stimulated for vibration, with the pickup and exciter delicately placed to ensure stable contact between the probe and the specimen. Due to the specimen’s uneven surfaces, a thin Vaseline layer was applied for optimal contact. A minimum of three measurements were required, with a ±0.5% tolerance between any two data points. The freeze–thaw cycle experiment was carried out in accordance with the specification of the “Standard Test Methods for Long-Term Performance and Durability of Ordinary Concrete” [23]. A KDR-V9 Concrete Rapid Freeze–Thaw Cycler was used to conduct the test, and the procedure of freeze–thaw cycle test is shown in Figure 2.

## 3. Results and Discussion

### 3.1. Experimental Phenomenon

**Figure 3:**
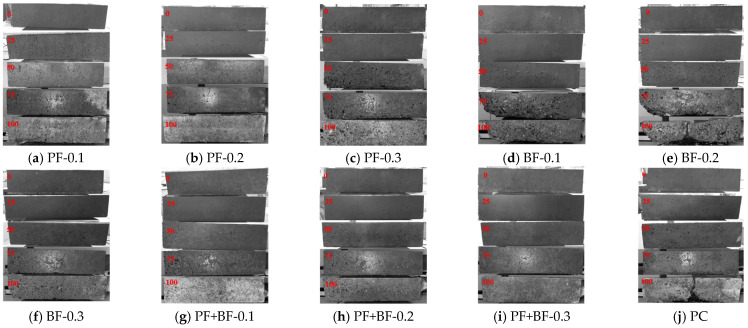
Apparent damage of Gobi aggregate concrete after different freeze–thaw cycles (the red font numbers represent the number of freeze–thaw cycles).

In order to facilitate the observation of the apparent damage to the specimens after freeze–thaw cycles, grayscale processing was applied to the images, as shown in Figure 3. From the Figure, the following can be observed:

1. The surfaces of specimens that have not undergone freeze–thaw cycles are relatively smooth, with relatively small pores.
2. After 25 freeze–thaw cycles, the surfaces of the specimens become rough, but no surface spalling is observed. There is no significant difference in surface roughness and pore count among concrete specimens with different types and amounts of fibers. This indicates that the freeze–thaw damage to concrete in the early cycles is not significantly influenced by the presence of fibers but is determined by the manufacturing process and initial defects in the specimens.
3. After 50 freeze–thaw cycles, the surface of the specimens becomes rougher, and the number of pores increases. Except for sporadic spalling at the edges of the PF0.3 specimen, no spalling is observed in the other specimens. Compared to the plain concrete group, the addition of fibers improves the apparent damage to varying degrees. The improvement effect, from highest to lowest, is as follows: polypropylene–basalt fibers, polypropylene fibers, and basalt fibers.
4. After 75 freeze–thaw cycles, the degree of apparent damage increases significantly. Compared to plain concrete, polypropylene fibers and mixed fibers can improve the apparent damage, but basalt fibers show noticeable spalling at the edges in the 0.1% and 0.2% content groups, likely due to uneven fiber mixing.
5. After 100 freeze–thaw cycles, the apparent damage further deteriorates. Plain concrete and 0.2% basalt fiber concrete experience extensive mortar and aggregate spalling with fractures. Polypropylene fiber concrete and the remaining basalt fiber concrete exhibit increases in the number and size of pores, along with spalling of the mortar layer at the edges. Polypropylene–basalt fiber concrete shows minimal changes in appearance, indicating that the improvement in anti-spalling performance from highest to lowest is as follows: polypropylene–basalt fibers, polypropylene fibers, and basalt fibers. At this point, all fiber-reinforced concrete specimens show exposed fibers on the outer surface.

In conclusion, the addition of fibers not only enhances the freeze resistance of concrete but also mitigates surface damage. The reason can be attributed to the fiber’s toughening and crack resistance effect, which ensures the integrity of the concrete and reduces the peeling of mortar and aggregate on the surface of the specimen.

### 3.2. Mass Loss Rate

**Figure 4:**
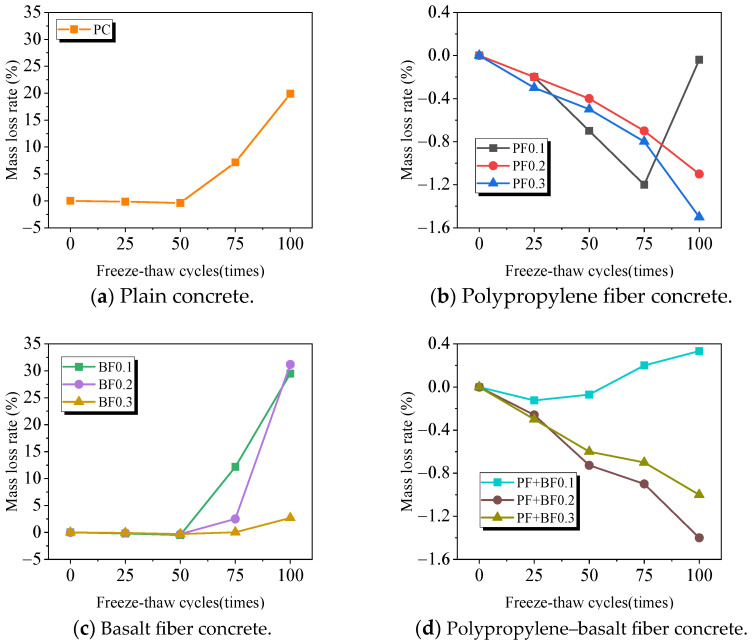
Mass loss rate of Gobi aggregate concrete after freeze–thaw cycles.

The freeze–thaw cycles of concrete primarily manifest as internal damage fatigue, accompanied by the spalling of cement mortar and coarse aggregates on the specimen surface. The mass loss rate serves as the primary indicator reflecting the external mortar spalling. The mass loss rate is calculated according to Equation (1). Figure 4 illustrates the mass loss rates of fiber-reinforced concrete under the influence of freeze–thaw cycles, taking the average values of three specimens in each group.

(1) $$\Delta W_{n}=\frac{W_{0}-W_{n}}{W_{0}}$$

where *W* represents the mass of concrete before freeze–thaw cycles, in kilograms (kg); *W_n_* represents the mass of the specimen after freeze–thaw cycles, in kilograms (kg); and Δ*W_n_* represents the mass loss rate (%) after freeze–thaw cycles.

As depicted in Figure 4, for plain concrete (see Figure 4a), the mass shows a slight increase before 50 freeze–thaw cycles, and a rapid increase in mass loss after 75 freeze–thaw cycles. For polypropylene fiber concrete (see Figure 4b), when the fiber content is 0.1%, the mass shows a small increase before 75 freeze–thaw cycles, and mass loss occurs after 100 freeze–thaw cycles. When the fiber content is 0.2% and 0.3%, the mass increases with the number of freeze–thaw cycles. In the case of basalt fiber concrete (see Figure 4c), the mass of specimens with 0.1% and 0.2% fiber content show slight increases before 50 freeze–thaw cycles, followed by a rapid increase in mass loss after 50 freeze–thaw cycles, with changes ranging from −0.5% to 29.5% and −0.3% to 31.2%, respectively. The trend for the concrete with 0.3% fiber content is similar but with a smaller magnitude within the range of −0.3% to 2.7%. For the polypropylene–basalt fiber concrete (see Figure 4d), the mass of specimens with 0.1% fiber content shows a slight increase before 50 freeze–thaw cycles, followed by a small decrease after 50 cycles. When the fiber content is 0.2% and 0.3%, the mass increases with the number of freeze–thaw cycles, with an overall change within 1.5%. This can be explained by the following: the clay minerals in Gobi aggregates result in higher water absorption [20]; in the early stages of the freeze–thaw cycle, the damage due to freeze–thaw, caused by the mortar and aggregate spalling phenomenon, is relatively minor. However, instead of its internal pores cracking due to the harmful pressure generated by the heat and cold cycle, it results in the production of new pore cracks in the concrete while expanding the internal pore cracks in the original ones. These pore cracks gradually absorb a large amount of water from the environment, causing the specimen to increase in size due to water absorption, exceeding the quality loss caused by aggregate spalling, thus leading to a weight gain in the specimen [24]. With the increase in the number of freeze–thaw cycles, the mass loss rate of plain concrete and concrete with a basalt fiber dosage of not more than 0.2% increased rapidly, indicating that the concrete containing gobstopper material sustained severe freeze–thaw damage. With the increase in the number of freeze–thaw cycles, the mass loss rate of concrete without fiber reinforcement and that with a basalt fiber content not exceeding 0.2% exhibits a rapid increase, indicating severe freeze–thaw damage to the Gobi concrete.

In summary, it is evident that the freeze-–thaw cycle numbers are the highest for concrete with solely polypropylene fiber, followed by concrete with a combination of polypropylene and basalt fibers. Basalt fibers achieve a similar effect to the combination when the content is 0.3%, indicating that an appropriate amount of fiber incorporation can mitigate the mass loss of Gobi aggregate concrete and improve its frost resistance after undergoing freeze–thaw cycles.

### 3.3. Relative Dynamic Elastic Modulus

In the freeze–thaw environment, concrete undergoes continuous fatigue damage, leading to a decline in frost resistance. The dynamic elastic modulus reflects the extent of internal damage, thereby indicating the specimen’s damage changes, making it the most commonly used indicator for the non-destructive testing and assessment of concrete. The relative dynamic elastic modulus is calculated according to reference [24], as shown in Equation (2):

(2) $$P_{n}=\frac{E_{n}}{E_{0}}\times 100\%$$

where *P_n_* represents the relative dynamic modulus of elasticity of concrete after *N* freeze–thaw cycles, expressed as a percentage; *E_n_* denotes the dynamic modulus of elasticity after *N* freeze–thaw cycles, measured in MPa; and *E*_0_ stands for the dynamic modulus of elasticity after 0 freeze–thaw cycles, also measured in MPa.

**Figure 5:**
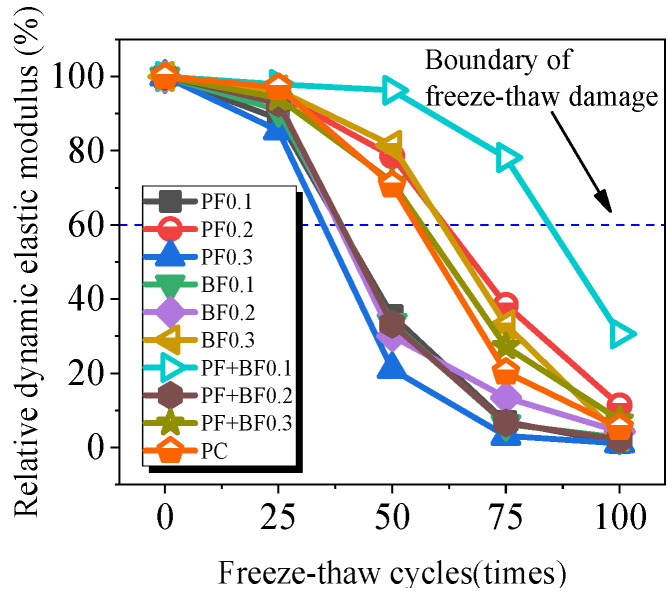
Relative dynamic elastic modulus of Gobi concrete after freeze–thaw cycles.

**Figure 6:**
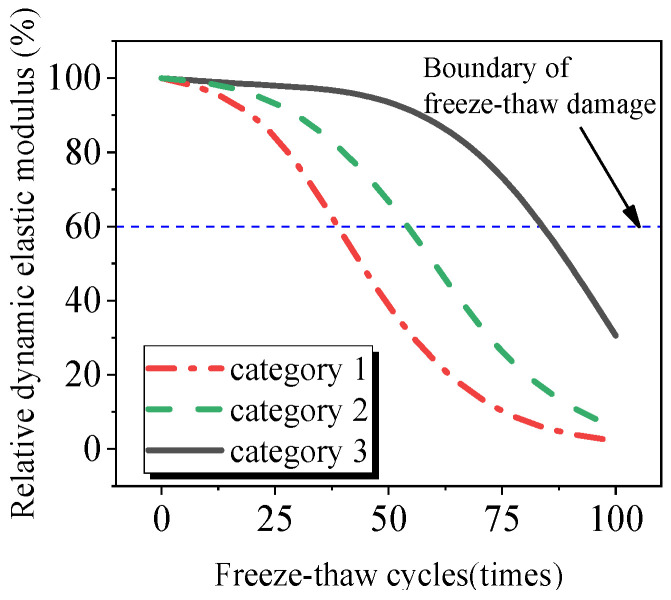
Classification of freeze–thaw cycles and relative dynamic elastic modulus relationship curve.

Figure 5 shows the relationship curve between the relative dynamic elastic modulus of Gobi concrete and the number of freeze–thaw cycles. For ease of analysis, based on the stopping criteria for concrete freeze–thaw cycle testing specified in the code of “Standard Test Methods for Long-Term Performance and Durability of Ordinary Concrete” (GB/T50082-2009), the relationship curve between the relative dynamic elastic modulus and the number of freeze–thaw cycles can be divided into three categories (see Figure 6): Category 1: Gobi aggregate concrete with polypropylene fiber contents of 0.1% and 0.3%, basalt fiber contents of 0.1% and 0.2%, and mixed fiber contents of 0.2%; Category 2: plain concrete, concrete with 0.2% polypropylene fiber content, concrete with 0.3% basalt fiber content, and concrete with a mixed fiber content of 0.3%; Category 3: concrete with a mixed fiber content of 0.1%.

According to Figure 5, it can be observed that after 25 freeze–thaw cycles, the relative dynamic elastic modulus of specimens in Category 1 is lower than that of plain concrete, while the difference in the relative dynamic elastic modulus between Category 2 and Category 3 fiber-reinforced concrete specimens and plain concrete is not significant. This indicates that fibers in Category 1 specimens lead to increased internal damage, thereby reducing frost resistance. After 50 freeze–thaw cycles, the relative dynamic elastic modulus of Category 1 specimens decreases by 21.1% to 35.5%, with the decrease in Category 2 specimens being smaller than in plain concrete and Category 3 specimens only decreasing by 3.7%. After 75 freeze–thaw cycles, the relative dynamic elastic modulus of Category 2 specimens decreases by 20.3% to 38.4%, but the decrease in the dynamic elastic modulus of fiber-reinforced concrete is smaller than that of plain concrete, indicating that fibers slow down internal concrete damage. The relative dynamic elastic modulus of Category 3 specimens decreases to 78.1%, demonstrating good frost resistance. After 100 freeze–thaw cycles, the decrease in dynamic elastic modulus for Category 1 and Category 2 specimens slows down, and Category 3 specimens decrease to 30.6%, meeting the termination requirements of the experiment. The reason can be explained as follows: on one hand, the incorporation of fibers can modify the microstructure of concrete by reducing pores and cracks, enhancing its compactness and uniformity, thereby improving its frost resistance [15]; on the other hand, fibers can block the capillaries of concrete, decreasing its water absorption rate and affecting the water distribution and crystallization process during freeze–thaw cycles, ultimately leading to a decrease in the frost resistance of concrete [25].

Within the scope of the experiment, the optimal improvement in the freeze–thaw resistance of Gobi concrete was achieved when the polypropylene fiber content, basalt fiber content, and the combined polypropylene–basalt fiber content were 0.2%, 0.3%, and 0.1%, respectively. It is worth noting that the improvement effect of the mixed fibers was superior to that of the single fiber, This can be explained as follows: when polypropylene fiber and basalt fiber are mixed, a “positive hybrid effect” is generated, which fully leverages their individual advantages and creates a more intricate network structure within the concrete; this intricate network structure effectively hinders crack propagation, thus enhancing the frost resistance of Gobi concrete.

## 4. Freeze–Thaw Damage Deterioration Model of Fiber-Reinforced Gobi Concrete

The damage to concrete caused by freeze–thaw cycles gradually weakens from the outer layer to the inner layer, and the change in the longitudinal wave propagation speed is relatively small. Therefore, using the relative dynamic elastic modulus to define freeze–thaw damage is more reasonable [26] and provides a better evaluation of the damage extent. Based on the changing pattern of the relative dynamic elastic modulus, this paper establishes a quadratic polynomial freeze–thaw damage model according to concrete freeze–thaw damage mechanics. It predicts the service life of fiber-reinforced Gobi aggregate concrete in northern regions.

According to the definition of damage mechanics, the concrete damage degree *D_E_* is defined as follows:

(3) $$D_{E}=1-\frac{E_{n}}{E_{0}}$$

Based on Equation (3), the freeze–thaw damage degree (*D_E_*) of concrete is calculated. The freeze–thaw damage model is fitted using Origin software (2018) in quadratic form, as shown in Equation (4):

(4) $$D_{E}=a+bN+cN^{2}$$

In the equation, *N* represents the number of cycles, and *a*, *b*, and *c* are coefficients to be determined through fitting.

**Figure 7:**
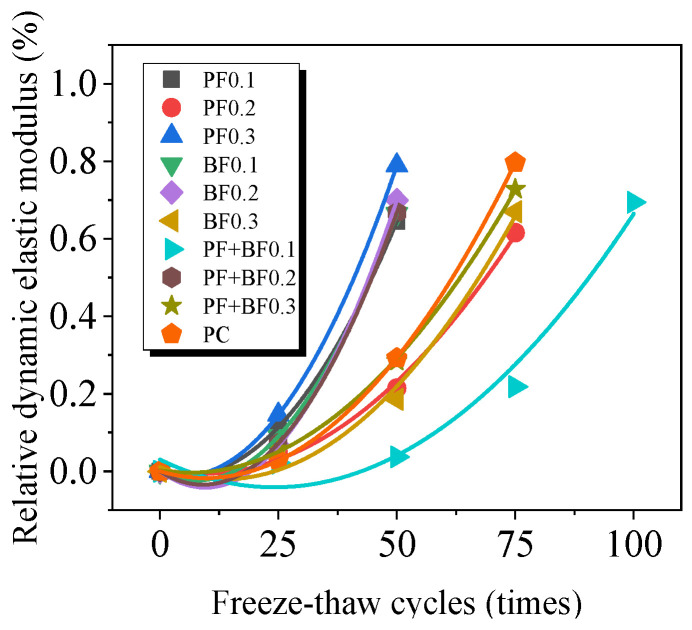
Fitting curve of freeze–thaw damage degree of Gobi aggregate concrete under freeze–thaw cycles.

Figure 7 shows the fitted curves of frost damage degree after different freeze–thaw cycles. Table 5 presents the fitting results of the quadratic damage model, with correlation coefficients ranging between 0.97 and 0.99. The high fitting precision suggests that the model can accurately predict the extent of freeze–thaw damage for this type of concrete.

**Figure 8:**
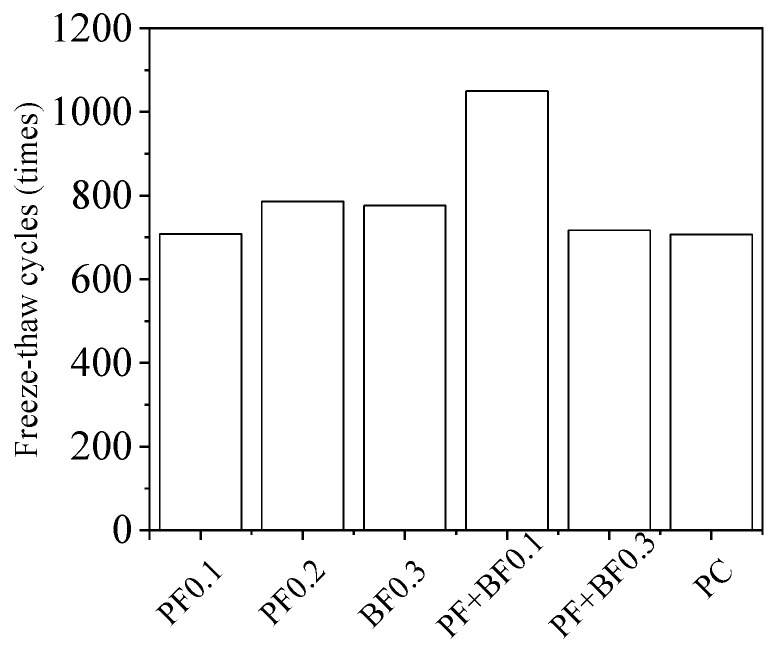
Prediction of freeze–thaw cycles for fiber-reinforced Gobi aggregate concrete.

To clarify the enhancement effect of fibers on the number of freeze–thaw cycles that Gobi aggregate concrete can withstand, the freeze–thaw damage model proposed in this section was employed for calculations. The specific method is as follows: firstly, calculate the number of freeze–thaw cycles corresponding to a relative dynamic elastic modulus reduction to 60% of the initial value. Then, based on the assumption that one indoor rapid freeze–thaw cycle is approximately equivalent to 10–15 natural environmental freeze–thaw cycles [27], this study calculated based on 12.5 cycles. The calculation results are shown in Figure 8.

As shown in Figure 8, the improvement effectiveness in descending order is as follows: 0.1% mixed fibers > 0.2% polypropylene fibers > 0.3% basalt fibers > 0.3% mixed fibers. Compared to the freeze–thaw cycle numbers of Gobi aggregate concrete under natural environmental conditions, these respective mixtures increased by 343 cycles, 79 cycles, 69 cycles, and 10 cycles. Considering all indicators, it is recommended to use a volume fraction of 0.1% mixed fibers to enhance the frost resistance of Gobi concrete. The reason can be attribute to the following: due to the differences in the physical and mechanical properties of polypropylene fibers and basalt fibers, the two types of fibers play an inhibitory role in different stages of crack development in concrete [9]. Therefore, the anti-freezing performance of mixed-fiber concrete is superior to that of concrete mixed with a single fiber. However, when the volume of mixed fibers is excessively large, it diminishes the uniformity of fiber dispersion, which compromises the adhesion between the fibers and the concrete matrix, ultimately resulting in a decrease in the anti-freezing performance of the concrete.

## 5. Conclusions

(1) Incorporating an appropriate fiber content can mitigate the loss of mass in Gobi concrete and improve its frost resistance after freeze–thaw cycles. Within the experimental range, the mass of Gobi concrete rapidly increased after 75 freeze–thaw cycles, reaching a maximum loss rate of 22.1%. The toughening and crack-resistance effects of fibers minimize the mass variations in Gobi concrete, resulting in a maximum mass loss rate of 2.7%.
(2) The impacts of different types of fibers on the relative dynamic elastic modulus of Gobi concrete varies. Within the scope of the experiment, for polypropylene-reinforced Gobi concrete, as the fiber content increases, the dynamic elastic modulus first increases and then decreases; for basalt fiber-reinforced Gobi concrete, as the fiber content increases, the dynamic elastic modulus also increases; for polypropylene–basalt fiber-reinforced Gobi concrete, as the fiber content increases, the dynamic elastic modulus decreases.
(3) There is an optimal fiber content for improving the freeze resistance of Gobi concrete. Too few fibers may not have a beneficial effect and may even exacerbate the defect of high mud content in Gobi aggregate, while excessive fiber content may obstruct the capillaries of the concrete, affecting water distribution and crystallization processes. The optimal volume contents of polypropylene fiber, basalt fiber, and polypropylene–basalt fiber in Gobi concrete are 0.2%, 0.3%, and 0.1%, respectively. Compared to the number of freeze–thaw cycles in Gobi concrete without fiber, these improvements resulted in increases of 343 cycles, 79 cycles, 69 cycles, and 10 cycles, respectively.
(4) A damage model based on a quadratic polynomial function was constructed using relative dynamic elastic modulus as the damage variable. The calculated values of this model match well with experimental results, accurately predicting the extent of freeze–thaw damage in fiber-reinforced Gobi concrete.

## Figures and Tables

**Table 1:** Basic physical properties of Gobi aggregate.

Category	Apparent Density/(kg/m^3^)	Bulk Density/(kg/m^3^)	Porosity (by Volume)/%	Fineness Modulus	Sediment Percentage/%	Clay Lump/%
Gobi coarse aggregate	2649.0	1436.9	45.8	/	2.9	/
Gobi fine aggregate	2605.0	1405.0	46.1	3.3	16.5	16.5

**Table 2:** Basic physical performance indicators of fibers.

Category	Length/mm	Diameter/μm	Tensile Strength/MPa	Elastic Modulus/GPa	Density/(g/cm^3^)
Polypropylene fiber	6.00	32.70	469.00	4.24	0.91
Basalt fiber	6.00	17.00	1050.00	7.60	2.65

**Table 3:** Relative percentage contents of mineral composition of clay in Gobi aggregate.

Sample	Relative Percentage Contents of Mineral Composition of Clay/%			
	Montmorillonite	Illite	Kaolinite	Chlorite
Gobi aggregate	31%	36%	14%	20%

**Table 4:** Mix proportions of fiber-reinforced Gobi aggregate concrete.

Specimen No.	Gobi Coarse Aggregate /(kg/m^3^)	Gobi Fine Aggregate /(kg/m^3^)	Cement /(kg/m^3^)	Fly Ash /(kg/m^3^)	Water /(kg/m^3^)	PCA/%	Polyethylene Glycol /(kg/m^3^)	Fiber Content/%
PF-0.1	1213.15	653.23	330.82	82.70	153.00	3.03	2.07	0.1
PF-0.2	1213.15	653.23	330.82	82.70	153.00	3.03	2.07	0.2
PF-0.3	1213.15	653.23	330.82	82.70	153.00	3.03	2.07	0.3
BF-0.1	1213.15	653.23	330.82	82.70	153.00	3.03	2.07	0.1
BF-0.2	1213.15	653.23	330.82	82.70	153.00	3.03	2.07	0.2
BF-0.3	1213.15	653.23	330.82	82.70	153.00	3.03	2.07	0.3
PF + BF-0.1	1213.15	653.23	330.82	82.70	153.00	3.03	2.07	0.1
PF + BF-0.2	1213.15	653.23	330.82	82.70	153.00	3.03	2.07	0.2
PF + BF-0.3	1213.15	653.23	330.82	82.70	153.00	3.03	2.07	0.3
PC	1213.15	653.23	330.82	82.70	153.00	3.03	2.07	/

Notes: PCA—water reducing agent; W/C—mass ratio of water to cementitious material; PF—polypropylene fiber; BF—basalt fiber; PC—plain concrete.

**Table 5:** Results of quadratic polynomial function fitting.

Specimen No.	*a*	*b*	*c*	*R* ^2^
PF0.1	4.3895 × 10^−4^	−0.0037	1.9026 × 10^−4^	0.9776
PF0.2	0.0057	−0.0026	1.4148 × 10^−4^	0.9972
PF0.3	0.0001	−0.0041	3.9808 × 10^−4^	0.9943
BF0.1	−3.7007 × 10^−17^	−0.0060	3.8648 × 10^−4^	0.9678
BF0.2	−7.4015 × 10^−17^	−0.0087	4.5416 × 10^−4^	0.9966
BF0.3	0.0112	−0.0048	1.7862 × 10^−4^	0.9912
PF + BF-0.1	0.0306	−0.0059	1.2272 × 10^−4^	0.9737
PF + BF-0.2	−1.3957 × 10^−16^	−0.0076	4.910 × 10^−4^	0.9989
PF + BF-0.3	0.0025	−0.0019	1.5414 × 10^−4^	0.9996
PC	4.3895 × 10^−4^	−0.0037	1.9026 × 10^−4^	0.9997

## Data Availability

Data are contained within the article.

## References

[1] Xia Q., Yang Y. (2010). Experimental study on the gobi soil filling for the subgrade of the second double-track in Lanzhou-Xianjiang railway. China Railw. Sci..

[2] Deng D., Liang Y., Huangfu F. (2021). Properties of gobi aggregate and sulfide-rich tailings cemented paste backfill and its application in a high-stress metal mine. Adv. Civ. Eng..

[3] Yang X., Xiao B., Gao Q., He J. (2020). Determining the pressure drop of cemented gobi sand and tailings paste backfill in a pipe flow. Constr. Build. Mater..

[4] Zhang T. (2003). Research on gobi aggregate concrete. Gansu Water Resour. Hydropower Technol..

[5] Farouk T. (2019). Study on key technologies of recycling gobi spoil materials in moncrete. Fujian Traffic Sci. Technol..

[6] Alafogianni P., Tragazikis I., Balaskas A., Barkoula N.-M. (2019). Structural properties and damage detection capability of carbon nanotube modified mortars after freeze-thaw. Materials.

[7] Dong W., Ji Y., Zhou M. (2023). Microstructure and service-life prediction models of aeolian sand concrete under freeze–thaw damage. Int. J. Pavement Res. Technol..

[8] Zhang P., Li Q. (2014). Freezing–thawing durability of fly ash concrete composites containing silica fume and polypropylene fiber. Proc. Inst. Mech. Eng. Part L J. Mater. Des. Appl..

[9] He W., Kong X., Fu Y., Zhou C., Zheng Z. (2020). Experimental investigation on the mechanical properties and microstructure of hybrid fiber reinforced recycled aggregate concrete. Constr. Build. Mater..

[10] Li W., Ji W., Wang Y., Liu Y., Shen R.-X., Xing F. (2015). Investigation on the mechanical properties of a cement-based material containing carbon nanotube under drying and freeze-thaw conditions. Materials.

[11] Małek M., Łasica W., Kadela M., Kluczyński J., Dudek D. (2021). Physical and mechanical properties of polypropylene fibre-reinforced cement–glass composite. Materials.

[12] Xu C., Huang S., Li H., Li H., Li Z., Lian H., Li Z. (2021). Damage of mechanical properties of basalt Fiber reinforced concrete under salt freezing. Bull. Chin. Ceram. Soc..

[13] Wang D., Ju Y., Shen H., Xu L. (2019). Mechanical properties of high performance concrete reinforced with basalt fiber and polypropylene fiber. Constr. Build. Mater..

[14] Yan W., Niu F., Wu Z., Niu F., Lin Z., Ning Z. (2016). Mechanical property of polypropylene fiber reinforced concrete under freezing-thawing cycle effect. J. Traffic Transp. Eng..

[15] Richardson A.E., Coventry K.A., Wilkinson S. (2012). Freeze/thaw durability of concrete with synthetic fibre additions. Cold Reg. Sci. Technol..

[16] Ren J., Lai Y. (2021). Study on the durability and failure mechanism of concrete modified with nanoparticles and polypropylene fiber under freeze-thaw cycles and sulfate attack. Cold Reg. Sci. Technol..

[17] Xie G., Shen X., Liu J. (2021). Frost resistance and damage degradation model of basalt fiber regenerated concrete. Compos. Sci. Eng..

[18] Xue Q., Zhang J., He J., Ramze T.J. (2016). Experimental study of fracture properties for basalt-fiber-reinforced concrete. J. Harbin Eng. Univ..

[19] Zhang J., Guan Y., Fan C., Cao G., Liu J. (2024). Experimental and theoretical investigations on the damage evolution of basalt fiber reinforced concrete under freeze-thaw cycles. Constr. Build. Mater..

[20] Li C., Li J., Tuerdimaimaiti M., Liao H., Chen Z. (2024). Investigation on physical properties and compressive strength of gobi aggregate concrete. Concrete.

[21] Powers T.C. (1945). A working hypothesis for further studies of frost resistance of concrete. J. Proc..

[22] (2011). Specification for Mix Proportion Design of Ordinary Concrete.

[23] (2009). Standard for Test Methods of Long-Term Performance and Durability of Ordinary Concrete.

[24] Zhang P. (2023). Frost Resistance of Fiber Concrete in Tibet Plateau Area Experimental Study on Durability Resistance. Master’s Thesis.

[25] Xiao Q., Hao S., Ning X. (2018). Experimental study on frost resistance of hybrid fiber reinforced concrete. Concrete.

[26] Zhang G., Geng T., Lu H. (2021). Damage model of desert sand fiber reinforced concrete under freeze-thaw cycles. Bull. Chin. Ceram. Soc..

[27] Yang H., Lin Z., Yu Y. (2023). Research on frost resistance and damage degradation model of basalt fiber reinforced concrete. Water Conserv. Constr. Manag..

